# The Underestimated Impact of Hashimoto Thyroiditis on Thyroid Papillary Carcinoma

**DOI:** 10.1007/s13304-024-01854-y

**Published:** 2024-04-30

**Authors:** Ahmet Tarik Harmantepe, Kayhan Ozdemir, Zulfu Bayhan, Belma Kocer

**Affiliations:** 1 Faculty of Medicine, Department of Gastrointestinal Surgery, Sakarya University, Sakarya, Turkey; 2 Faculty of Medicine, Department of General Surgery, Sakarya University, Sakarya, Turkey

**Keywords:** Papillary thyroid cancer, Hashimoto’s thyroiditis, Lymphocytic infiltration

## Abstract

It is stated that Hashimoto’s Thyroiditis (HT) is a risk factor for the development of Papillary Thyroid Cancer (PTC). However, the effect of HT on the coexistence of HT and PTC is still controversial. In this study, our aim is to investigate the effect of the presence of HT on clinicopathological data in patients with PTC. All 356 patients whose pathology was reported as PTC who were operated between 2015 and 2023 were included in the study. PTC patients were divided into 2 groups as those with and without HT. The effect of HT association on clinicopathological features was investigated. In 356 PTC patients, the rate of HT was 31.2%. PTC patients with HT had less multifocality (*p* < 0.05), more lymph node metastases (LNM) (*p* < 0.01) compared to PTC patients without HT. The presence of HT did not affect the bilaterality of the tumor, tumor diameter, lymphovascular invasion, or capsule invasion. While multifocality was observed less frequently in PTC patients with HT, lymph node metastasis rates were higher.

## Introduction

Hashimoto’s Thyroiditis (HT) is the most common autoimmune thyroid disease [1]. HT is also called chronic lymphocytic or autoimmune thyroiditis. Its incidence is 0.3–1.5 per 1000, and it is more common in women (5–20 times) [2]. Hypothyroidism, enlargement of the thyroid gland with inflammatory cells, and atrophy due to autoimmunity occur in HT. HT is a risk factor for the development of Papillary Thyroid Cancer (PTC) [3]. Recently, studies have also focused on the impact of HT on the prognosis of patients with HT-associated PTC.

Many etiological and epidemiological studies have investigated the relationship between PTC and HT. Most of them have argued that HT can counteract the progression of PTC. Some authors have shown that HT and PTC are associated with pathological factors, such as small tumor diameter and early stage, indicating reduced tumor aggressiveness [4]. Some researchers report that PTC patients with lymphocytic thyroiditis tend to have less lymph node metastases (LNM) and better prognoses [5]. These relationships are plausible because lymphocytic infiltration is conceivable in enhancing antitumor immunity. In contrast, other studies have shown that PTC co-existing with HT is more likely to be bilateral and multifocal [6, 7]. Some studies have revealed that multifocal cases are associated with an increased incidence of central lymph node metastases (CLNM) [8]. However, other studies did not find a difference between unifocal and multifocal PTC with HT [9]. In general, the relationship of lateral lymph node metastases (LNM) and multifocal PTC with HT is not fully comprehended.

Our aim in this study is to investigate the effect of HT on tumor progression in patients with PTC.

## Materials and methods

It was approved by the ethics committee. The study was designed retrospectively. All 356 patients who underwent thyroidectomy for different indications in our center between January 2015 and March 2023 and whose PTC diagnosis was confirmed histopathologically were included in the study. Histopathological examination was performed with immunohistochemical stains (HBME-1, Galectin-3, CD56, and CD34) on 5 mm sections. All PTC patients were evaluated in terms of various clinicopathological features, such as age, gender, tumor diameter, presence of chronic lymphocytic thyroiditis (CLT), extra-thyroidal extension, lymphovascular invasion (LVI), multifocality, number of foci, bilaterality, lymph node metastasis, number of lymph node metastases, and central or lateral compartment involvement. Patients with CLT in histopathological examination were grouped as HT( +) and patients without CLT were grouped as HT( – ). Afterwards, clinicopathological features of PTC with and without HT were compared.

Clinical assessments for all patients included ultrasonic examination of the thyroid and ultrasound-assisted fine needle aspiration biopsies (FNAB). The surgical indications of the patients were determined according to the 2015 guidelines of the American Thyroid Association. In patients with high suspicion of malignancy, thyroid FNAB was performed based on ultrasound examinations before thyroidectomies. Therapeutic central lymph node dissection was performed in patients with confirmed preoperative lymph node involvement, and prophylactic bilateral central neck dissection was performed in patients with papillary carcinoma without lymph node involvement but with a lesion larger than 4 cm or extra-thyroidal invasion. Lateral lymph node dissection was performed in patients who reported malignant LN metastasis with preoperative FNAB in the lateral compartment.

Data were analyzed using the SPSS 23.0 program. *p* < 0.05 was considered significant. The Kolmogorov–Smirnov test was applied to check whether the parametric data followed a normal distribution. Numerical data that comply with normal distribution are shown with a mean (± standard deviation), numerical data that do not comply with normal distribution are shown with median (minimum–maximum), and categorical data are shown with a number (%). The chi-square test was used to analyze the significance between the categorical variables.

## Results

### General features of patients with PTC

A total of 356 patients whose pathology results were reported as PTC were included in the study. Of these patients, 77.5% (*n* = 276) were female and 22.5% (*n* = 80) were male. The mean age was 46.95 (± 13.1). The median tumor diameter was 1.42 (0.10–8.50) cm. HT association was present in 111 (%31.2) of the patients. The rate of extra-thyroidal invasion was 30.3%, LVI rate was 12.1%, multi-focality rate was 28.4%, bilateral incidence was 12.1%, LNM rate was 14.3%. (Table 1) There was no patient with preoperative hypothyroidism.

**Table 1:** Clinicopathological features of all patients and the effect of HT on these features

Clinicopathological parameters	*N* (%)	HT – *N* (%)	HT + *N* (%)	*p* value
Number of patients		245 (%68,8)	111 (%31,2)	
*Age*				
< 55	248 (%69,7)	170 (%69,4)	78 (%70,3)	*p* = 0,86
> 55	108 (%30,3)	75 (%30,6)	33 (%29,7)	
*Gender*				
Female	276 (%77,5)	194 (%79,2)	82 (%73,9)	*p* = 0,26
Male	80 (%22,5)	51 (%20,8)	29 (%26,1)	
*Tumor size*				
< 2 cm	275 (%77,2)	192 (%78,4)	83 (%74,8)	*p* = 0,37
2–4 cm	54 (%15,2)	33 (%13,5)	21 (%18,9)	
> 4 cm	27 (%7,6)	20 (%8,2)	7 (%6,3)	
*Tumor Uni-/Bilateralite*				
Unilateral	313 (%87,9)	214 (%87,3)	99 (%89,2)	*p* = 0,62
Bilateral	43 (%12,1)	31 (%12,7)	12 (%10,8)	
*Multifocality*				
Absent	255 (%71,6)	167 (%68,2)	88 (%79,3)	*p* < 0,05
Present	101 (%28,4)	78 (%31,8)	23 (%20,7)	
*Number of focus*				
1 focus	256 (%71,9)	168 (%68,6)	88 (%79,3)	*p* = 0,11
2 foci	69 (%19,4)	53 (%21,6)	16 (%14,4)	
> 2 foci	31 (%8,7)	24 (%9,8)	7 (%6,3)	
*LNM*				
Absent	306 (%85,7)	219 (%89,4)	87 (%78,4)	*p* < 0,01
Present	50 (%14,3)	26 (%10,6)	24 (%21,6)	
*LNM location*				
CLNM only	24 (%48)	12 (%46,2)	12 (%50)	*P* = 0,78
CLNM + LLNM	26 (%52)	14 (%53,8)	12 (%50)	
*LVI*				
Absent	313 (%87,9)	220 (%89,8)	93 (%83,8)	*p* = 0,10
Present	43 (%12,1)	25 (%10,2)	18 (%16,2)	
*Extra-thyroidal invasion*				
Absent	248 (%69,7)	172 (%70,2)	76 (%68,5)	*p* = 0,74
Present	108 (%30,3)	73 (%29,8)	35 (%31,5)	

### Comparison of clinicopathological features between patients with PTC with and without HT

Clinicopathological features of patients with PTC with and without HT were compared. There were 111 (31.2%) patients with HT and 245 (68,8%) patients without HT. Gender, age, bilaterality, multifocality, tumor size, capsule invasion, lymphovascular invasion, CLNM, and LLNM rates were compared in patients with and without HT (Table 1). Tumor diameter, number of focuses, lymphovascular invasion, and capsule invasion did not differ significantly in the results. The rate of multifocality was significantly lower in HT patients (*p* < 0.05). However, the LNM rate was significantly higher in HT patients (*p* < 0.01). From patients have LNM, the rate of CLNM was 50% (*n* = 12) in patients with HT and 46.2% (*n* = 12) in patients without HT. The rate of LLNM was 50% (*n* = 12) in patients with HT, while it was 53.8% (*n* = 14) in patients without HT. (Table 1).

## Discussion

HT is the most common form of autoimmune thyroid disease [10]. The incidence of HT is about 0.3–1.5 cases per 1000 people per year [11]. Both cellular and humoral immunity play a role in its pathogenesis. In autoimmune thyroid disease, T cells migrate from the periphery to the thyroid gland and actively participate in the autoimmune process. Studies have shown defects in the T suppressor cell response to thyroid-specific antigens in autoimmune hypothyroidism and antigen-specific T suppressor dysfunction in the pathogenesis of the disease [12]. In autoimmune thyroid diseases, CD8 + T cells are detected against both thyroid peroxidase enzyme (TPO) and thyroglobulin (TG) and mediate gland destruction [13]. In addition, environmental factors, such as excessive iodine intake, various viral infections and drugs played a role in the etiology of HT [14]. Some studies have revealed that the disease develops due to increased T cell activation and determines the relationships between certain tissue groups, such as human leukocyte antigen (HLA), DR3, DR4, and DR5 [15]. Moreover, many genetic factors regulating immunological reactions have been held responsible for the emergence of the disease and this concept has been supported by many studies [16, 17]. The disease often leads to hypothyroidism, which is characterized by T3 and T4 deficiency and elevated TSH levels. In addition, anti-TG and especially TPO-Ab elevation helps in diagnosis [18]. On ultrasonography, there are hypoechoic solid nodules and heterogeneous thyroid parenchyma structure. [19]

There are many studies examining the relationship between autoimmune diseases and cancer [20–22]. The development of cancer secondary to autoimmune diseases may result from various mechanisms: chronic inflammation and tissue damage caused by autoimmunity, failure to clear oncogenic viral infections, and long-term immunosuppressive therapies for rheumatic disease. [23–25]

One of the most common endocrine malignancies is PTC and the incidence of this condition has increased rapidly in recent years [26]. The relationship between PTC and HT is often the subject of discussion. The relationship between PTC and HT was first described by Dailey in 1955 [27]. It has been frequently reported in the literature that HT is a risk factor for the development of PTC [3, 28]. However, several retrospective and prospective studies have not found a correlation between these two diseases [29, 30]. Many studies have focused on this relationship. But the debates are still going on.

Some studies have reported conflicting results regarding the biological behavior of PTC in the presence of HT. However, although all of these studies reported different histopathological rates, they concluded that PTC had a better prognosis in the presence of HT. In the study of Liang et al. in cases with PTC accompanied by HT, smaller tumor size, higher multifocality, and better PTC prognosis were concluded compared to those without HT [4]. Although multifocality was higher, it was interpreted as a good prognosis because of less lymph node metastasis. In a study by Zhu et al. they concluded a better PTC prognosis due to higher multifocality but lower CLNM in cases with HT, but they found no significant difference in LLNM [5]. In the meta-analysis conducted by Mao et al. when they investigated the factors affecting lymph node metastasis in PTC, it was found that HT had no effect [31]. In a study by Zhang et al. they concluded that PTC patients with HT have a better PTC prognosis due to smaller tumor diameter, less invasion, less lateral neck metastasis, and less multifocality [3]. Although multifocality and tumor size were reported at different rates in these studies, lymph node metastasis rates, which is an important factor affecting prognosis, were found to be lower in patients with HT. In our study, no significant difference was observed between PTC with HT and PTC without HT in terms of tumor size, lymphovascular invasion, and capsule invasion. Multifocality was detected less in cases with HT, but contrary to the literature, more LNM rates were found in patients with HT in our study. Perhaps theoretically, the fact that TSH is a growth factor for differentiated thyroid cancer supports our study. However, none of our patients had preoperative hypothyroidism. However, we do not know how long the patients were exposed to high TSH before.

Therapeutic lymph node dissection is recommended for patients with CLNM or LLNM detected by preoperative ultrasonography or physical examination. However, there is no consensus on the indications for prophylactic lymph node dissection. While the American Thyroid Association (ATA) suggests that prophylactic central lymph node dissection should be performed in PTC patients with stage T3 or T4, The National Comprehensive Cancer Network (NCCN) guidelines recommend prophylactic central lymph node dissection in patients with PTC under the age of 15 or over the age of 45, with a tumor larger than 4 cm or with extra-thyroidal invasion [32, 33]. In the light of our results, considering that LNM rates are higher in PTC patients with HT, we think that the option of performing prophylactic lymph node dissection should also be considered in this patient group. However, it will be possible to reach a more precise result with higher volume studies.

Since our study was retrospective and single-center, our patient number and access to clinical, radiological and biochemical data were limited. However, according to the data we can obtain, LNM should be considered in patients with PTC accompanied by HT, and treatment planning should be done accordingly.

## Conclusion

As a result, LNM rates were higher in PTC patients with HT, unlike similar studies in the literature. This result adds originality to our study. Prospective studies with a large number of cases are needed in this regard.

## Data Availability

The datasets analyzed during the current study are available from the corresponding author upon reasonable request.
